# Long-term outcomes and predictors of recurrent aortic regurgitation after aortic valve-sparing and reconstructive cusp surgery: a single centre experience

**DOI:** 10.1186/s13019-019-1019-3

**Published:** 2019-11-12

**Authors:** Dainius Karciauskas, Vaida Mizariene, Povilas Jakuska, Egle Ereminiene, Jolanta Justina Vaskelyte, Irena Nedzelskiene, Sarunas Kinduris, Rimantas Benetis

**Affiliations:** 1 Department of Cardiac, Thoracic and Vascular Surgery, Medical Academy, Lithuanian University of Health Sciences, A. Mickeviciaus g. 9, 44307 Kaunas, LT Lithuania; 2 Department of Cardiology, Medical Academy, Lithuanian University of Health Sciences, Kaunas, Lithuania; 3 Department of Dental and Oral Diseases, Lithuanian University of Health Sciences, Kaunas, Lithuania

**Keywords:** aortic cusp repair (ACR), aortic regurgitation (AR), aortic valve (AV), Effective height (eH), Transoesophageal echocardiography (TEE)

## Abstract

**Background:**

Aortic valve sparing surgery (AVS), in combination with aortic cusp repair (ACR), still raises many questions about the increased surgical complexity and applicability for patients with pure aortic valve regurgitation (AR). The aim of this study was to investigate our long-term outcomes and predictors of recurrent AR (> 2+) after AVS and reconstructive cusp surgery.

**Methods:**

We reviewed data of 81 patients who underwent AVS (a reimplantation technique) with concomitant ACR for AR and or dilatation of the aortic root at our institution during the period from April 2004 to October 2016. On preoperative echocardiography, the majority of the patients, 70 (86.4%) presented with severe AR grade (> 3+) and 28 (34.5%) of the patients had the bicuspid phenotype. Time to event analysis (long-term survival, freedom from reoperation, and recurrence of AR > 2+) was performed with the Kaplan–Meier method. Multivariate Cox regression risk analysis was performed to identify independent predictors of recurrent AR (> 2+). The mean follow-up was 5.3 ± 3.3 years and 100% complete.

**Results:**

The in-hospital (30-day) mortality rate after elective surgery was 1.2%. The overall actuarial survival rates were 92.9 ± 3.1% and 90.4 ± 3.9% at five and 10 years, respectively. Actuarial freedom from recurrent AR (> 2+) was 83.7 ± 4.5% within the cohort at five and 10 years. The cumulative freedom from all causes of cardiac reoperation was 94.2 ± 2.8% within the cohort at 10 years. Neither bleeding nor thromboembolic or permanent neurologic events were reported during follow-up. By multivariate analysis, independent predictors of reccurent AR (> 2+) were an effective height lower than 9 mm (p= 0.02) and intraoperative residual mild AR (p= 0.0001).

**Conclusions:**

AVS with ACR, combined in a systematic fashion, is a safe and reproducible option with low risk of long-term valve related events and normal life expectancy for patients with pure aortic regurgitation.

The competent aortic valve and effective height, not lower than 9 mm intraoperatively, are mandatory to achieve long-lasting AV competency.

## Introduction

Aortic valve-sparing (AVS) surgery associated with aortic cusp repair (ACR) has expanded the use of valve-sparing procedures in patients with complex aortic valve regurgitation (AR) to avoid aortic valve (AV) replacement and its inherent pitfalls [1]. However, AVS is a technically demanding procedure, and concerns regarding the increased additional surgical complexity and long-term outcomes associated with ACR are being addressed, particularly in high-risk patients with severe preoperative AR, eccentric jet, or different bicuspid phenotypes [2]. The concept of AVS and reconstruction of functional aortic root complex geometry with the reimplantation technique (David’s procedure) has been accepted systematically and used successfully by other aortic valve surgery groups with competitive long term outcomes [3–6]. However, there is great hesitation towards the AVS with ACR, as the long-term outcomes and predictors associated with cusp repair failure are not well defined and still controversial.

In this manuscript, we review our single institutional experience with AVS and primary ACR, focusing mainly on the long-term outcomes and predictors of recurrent AR (> 2+).

## Materials and methods

### Study population

Between April 2004 and October 2016, 92 consecutive adult patients underwent AVS surgery.

Of these, 81 patients underwent AVS with primary ACR for aortic valve regurgitation (AR) and or dilatation of the aortic root at our institution. Patients with expanded indications, such as severe progressive AR, bicuspid aortic valve (BAV), and aortic dissection were included in the present cohort. Patients were selected on the basis of preoperative echocardiographic findings only with pliable, good quality mobile aortic cusp tissue. Extensive structural cusp defects of the AV, such as lack of tissue (heavy fenestrations), extensive calcification, or restriction were considered unacceptable for AV preserving surgery and the patients that required the addition of a pericardial patch due to the inadequacy of the native cusp tissue (type III AR dysfunction) also were excluded. Preoperative, postoperative, and follow-up data were collected prospectively and analysed retrospectively. This investigation was performed in accordance with the Declaration of Helsinki and approved by the Regional Medical Research Ethics Committee of the Lithuanian University of Health Sciences (No.BE-2-34, the 12th of December 2016) and waived the need for individual informed consent due to the retrospective design of the study.

### Echocardiographic assessment

All selected patients completed detailed preoperative transthoracic and intraoperative transoesophageal echocardiographic (TEE) examination. Transthoracic echocardiographic images were obtained for all patients prior to discharge and at regular intervals (3, 6, and 12 months and annually thereafter) for living patients with native valves during the course of follow-up. The echocardiography protocol included the evaluation of AV anatomy (the number of cusps, fusions, and effective height), AV function (AR grade), dimensions of the aortic root, left ventricle (LV) end-diastolic diameter, and its systolic function (Table 1). The severity of AR was evaluated using a semiquantitative scale of colour flow Doppler echocardiography and graded as follows: absent, mild (1+), moderate (2+), or severe (≥ 3+). For a more comprehensive analysis of severity of AR, other quantitative parameters whenever possible were included (e.g., the width of the vena contracta). Echocardiographic parameters were assessed and reported according to the guidelines published by the European Association of Echocardiography [7].

**Table 1:** Preoperative clinical and echocardiographic characteristics of the study patients

Characteristics	Value
Age (years) Gender (male/female) BSA	50.8 ± 15.2 74 (91.3)/7 (8.6) 2.0 ± 0.2
NYHA class	
I/II III IV	46 (56.7) 33 (40.7) 2 (2.4)
Comorbidities	
AH CAD AF MI DM	57 (70.3) 24 (29.6) 13 (16) 2 (2.4) 2 (2.4)
Morphology	
BAV BAV type 1	28 (34.5) 21 (75)
Left ventricle	
LV EDDi (mm/BSA) LV EF (%)	28.6 ± 5.1 51.1 ± 8.1
Aortic root dimensions	
AVJ diameter (mm) Sinuses diameter (mm) STJ diameter (mm) Asc Ao diameter (mm)	28.5 ± 3.2 49.0 ± 7.7 44.0 ± 7.8 48.2 ± 9.6
AR grade	
0–1+ 2+ ≥ 3+	4 (4.9) 7 (8.6) 70 (86.4)

Values are expressed as mean ± SD or numbers (percentages)

*AF* atrial fibrillation, *AH* arterial hypertension, *AR* aortic valve regurgitation, *AscAo* ascending aorta, *AVJ* aortoventricular junction, *BAV* bicuspid aortic valve, *BSA* body surface area, *CAD* coronary artery disease, *DM* diabetus mellitus, *LV EDDi* left ventricle end diastolic diameter index, *LV EF* left ventricle ejection fraction, *MI* myocardial infarction, *NYHA New York Heart Association* functional classification*, STJ* sinotubular junction

### Follow-up

The clinical follow-up data were collected through either outpatient visits (institutional database and medical records) or by telephone interviews and completeness of the follow-up was 100%. The mean time of the clinical follow-up was 5.3 ± 3.3 years (range, 1.4–13.2 years). Follow-up echocardiographic examinations (at least > 1 year after the initial repair), were available in 86.4% (70) living patients free from interventions on their native aortic valve with a mean time 4.3 ± 3.5 years (range, 1.4–13.2 years). The primary outcomes of our study were long-term survival, freedom from reoperation on the AV, and the recurrence of AR > 2+. Postoperative adverse events were presented according to the criteria for the reporting of morbidity and mortality after cardiac valvular operations [8].

### Statistical analysis

Continuous variables with normal distribution are summarised as mean ± SD and data that did not exhibit a normal distribution, are summarised as median ± interquartile range (IQR), respectively. Continuous variables, depending on the normality of the distribution were analysed using the Student‘s t test (normally distributed data) or the Mann–Whitney U-test (non-normally distributed data). Categorical variables were analysed using the Chi-square test (Fisher’s exact test) and expressed as counts and percentages. Time to event analysis (overall survival rates, freedom from AV reoperation, and recurrence of AR > 2+) was estimated by the Kaplan–Meier method. All probability values less than (*p* < 0.05) was considered statistically significant.

The univariate analysis was used to identify potential preoperative and perioperative predictors (Table 3) associated with the development of recurrent AR (> 2+). All significant predictors (*p* < 0.02) were subsequently included and examined by a Multivariate Cox Proportional Hazards Analysis (Table 4). All analyses were conducted using the SPSS software version 23.0 (SPSS Inc., Chicago, Illinois). Graphs were constructed with the MedCalc Statistical Software version 14.8.1 (MedCalc Software bvba, Ostend, Belgium).

## Results

### Patients population

The mean age of the study population was 50.8 ± 15.2 (range, 18–81) years and 74 (91.3%) patients were male. On preoperative echocardiography, the majority of the patients 86.4% (70) presented with severe AR grade (> 3+) and 28 (34.5%) of the patients had the BAV phenotype. Other preoperative demographic patients characteristics (*New York Heart Association (NYHA)* functional class, prevelance of comorbidities and echocardiographic imaging data) are provided in Table 1.

### Surgical techniques

All patients underwent AVS with the reimplantation technique using the Valsalva graft (Gelweave Valsalva graft; Vascutek, Refrewshire, Scotland, UK). Our surgical strategy for AVS has been described previously [9]. Intraoperatively, the main criterium for final decision-making to proceed with AV sparing repair surgery was direct surgical inspection of the AV cusp tissue (quality, quantity, mobility) and confirmation of the findings in compliance with the preoperative TEE evaluation. Following a deep dissection of the aortic root and AV reimplantation into prosthesis, AV cusps were re-evaluated again for any dysfunction (symmetry and coaptation) and managed by the addition of ACR. ACR techniques performed at the time of AVS are guided by the “Repair Oriented Functional Classification of AR “[10] to achieve the increased coaptation (mid height of the sinuses of the Valsalva) and better AV competency (Table 2).

**Table 2:** Intraoperative data of the study patients

Characteristics	Value
Elective surgery Acute type A dissection Previous root surgery	78 (96.2) 3 (3.7) 3 (3.7)
Valsalva graft size (mm)	28.6 ± 2.2
Aortic cusp repair techniques	
Free margin central plication Free margin resuspension Triangular resection Shaving	68 (83.9) 14 (17.2) 12 (14.8) 4 (4.9)
Residual Aorta surgery	
AscAo/hemi-arch Full arch	9 (11.1) 2 (2.4)
Concomitant surgery	
MV repair TV repair CABG Other**	8 (9.8) 4 (4.9) 11 (13.5) 4 (4.9)
CPB time (min) AoC time (min)	130.0 (127–141.7) 98.0 (93–102)

Values are expressed as mean ± SD, numbers (percentages) or median (IQR)

Other** (atrial septal defect closure, pulmonary artery surgery)

*AoC* aortic cross clamp, *AscAo* ascending aorta, *CABG* coronary bypass grafting, *CBP* cardiopulmonary bypass, *MV* mitral valve, *TV* tricuspid valve

Of note, the majority of the patients who presented with cusp prolapse (type II AR dysfunction) were treated mainly using central-free margin plication of the cusp with 6–0 prolene and or in combination with free-margin resuspention using a running 7–0 Gore-Tex suture (W. L. Gore & Associates, Inc., Flagstaff, Ariz) as described elsewhere [11]. Triangular resection (including BAV type 1 patients) was performed with primary reapproximation using direct suture.

### Operative data

All patients with AVS and ACR were included in the present study, even patients who required more extensive and emergent surgery (previous cardiac surgery, concomitant procedures, and acute aortic syndromes). Primary ACR techniques used at the time of AVS were free-margin central plication in 68 (83.9%) patients, free-margin resuspension in 14 (17.2%) patients, triangular resection with direct suture in 12 (14.8%) patients, and shaving in 4 (4.9%) patients. Intraoperative data are summarised and presented in Table 2. Two patients (2.4%) required intraoperative reexploration of the AV and re-repair due to moderate AR after intraoperative evaluation by TEE. None of the patients left the operating room with greater than mild AR. All patients completed the initially planned AV repair sparing surgery without conversion to aortic valve replacement procedure. The ascending aorta (hemi-arch) and arch replacement procedures were performed in 11 (13.5%) patients with extensive aorta pathology. Twenty-seven (33.3%) patients had other concomitant cardiac procedures performed at the time of the AV sparing repair surgery, including mitral valve repair, coronary bypass grafting, and other (Table 2). The median cardiopulmonary bypass and cross-clamp times are presented in Table 2.

### Early outcomes

The in-hospital (30-day) mortality rate was 1.2% (1) in elective surgery (sepsis) and 2.4% (2) in emergency cases after surgery for acute type A dissection (perioperative brain injury and intraoperative type B dissection). No significant major valve-related events (structural valvular deterioration, endocarditis, thromboembolism, bleeding or permanent pacemaker implantation) were found during the course of hospital stay. Early re-exploration due to blood loss (coagulopathy related) was necessary for 17 (20.9%) patients. There were no postoperative permanent neurological events. At discharge, the echocardiographic evaluation was available for all patients and documented no more than mild AR (Table 2).

### Late follow-up

**Fig. 1:**
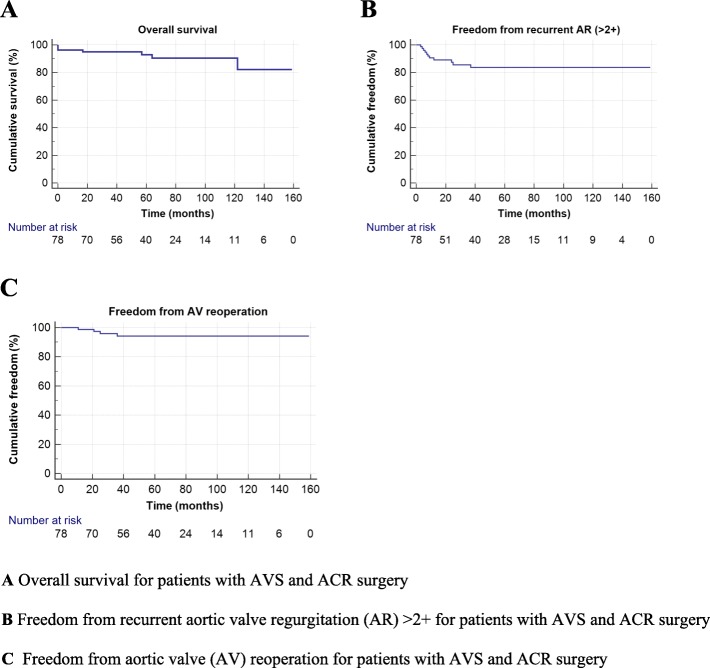
Kaplan–Meier curves in patients with aortic valve sparing surgery (AVS) and aortic cusp repair (ACR) surgery. **a** Overall survival for patients with AVS and ACR surgery. **b** Freedom from recurrent aortic valve regurgitation (AR) > 2+ for patients with AVS and ACR surgery. **c** Freedom from aortic valve (AV) reoperation for patients with AVS and ACR surgery

The overall acturial survival rates within the entire cohort were 92.9 ± 3.1% and 90.4 ± 3.9% at five years and 10 years, respectively (Fig. 1a). There were four late deaths; two cardiac related (congestive heart failure and sudden *unexplained* death after 17 and 122 months, respectively), and two from other non-cardiac causes (head trauma and cancer). At the latest follow-up contact, among living patients with native valves, the NYHA functional class I and II was in 88.5 and 11.4% patients, respectively. Freedom from recurrence more than moderate AR (> 2+) within the cohort was 83.7 ± 4.5% at five years and 10 years (Fig. 1b).

At the latest available echocardiographic follow-up, the AR grade was 0–1+ in 57 (73%) patients, AR gradre was 2+ in 10 (12.8%) patients, whereas 11 (14.1%) patients had more than moderate AR grade (> 2+). Of these, patients with an AR grade (> 2+), one patient died (congestive heart failure), three patients needed reoperation, and the remaining seven asymptomatic patients were being followed clinically and echocardiographically. Patients with AR grade (> 2+) characteristics are presented in Table 3.

**Table 3:** Characteristics of the patients with recurrent AR (> 2+)

Patien. Nr.	AV	Age (years)	SI	PreAR (3+)	AVJ (mm)	ACR technique	TEE AR + 1	eH (mm)	Mechanism	rAR (months)
1	TAV	54	–	1	29	FMR	1	3	Prolap	8
2	BAV	42	S1	1	29	RZ, FMR	1	10	Dehis	9
3	BAV	34	S1	1	31	RZ, FMR	1	6	Dehis	5
4	TAV	50	–	1	31	FMP,FMR	1	6.2	Restr	25
5	BAV	18	S1	1	27	FMP	0	6.1	Prolap	3
6	TAV	53	–	1	28	FMP	1	5.5	Prolap	24
7	BAV	48	S1	1	29	FMP	1	6	Restr	37
8	TAV	64	–	1	32	FMP	1	7	Prolap	6
9	BAV	33	S1	1	30	FMR	1	8	Dehis	4
10	TAV	52	–	1	27	FMP	1	8	Prolap	7
11	BAV	48	S0	1	29	FMP	1	10	Prolap	12

*ACR* aortic cusp repair, *AR* aortic regurgitation, *AV* aortic valve, *AVJ* aortoventricular junction, *BAV* bicuspid aortic valve, *Dehi* dehisence, *eH* effective height, *FMP* re- margin central plication, *FMR* free margin resuspension, *pAR* preoperative aortic regurgitation, *Prolap* prolapse, *rAR* recurrent aortic regurgitation, *Restr* Restriction, *RZ* triangular resection, *SI* Sievers type, *TEE* transoesophageal echocardiography

A multivariate Cox regression analysis identified intraoperative residual mild AR (HR 24.9; 95% CI 5.6–120; p= 0.0001) and an effective height lower than 9 mm (HR 5.1; 95% CI 1.3–19.1; p= 0.02) as independent significant predictors of recurrent AR(> 2+) at follow-up (Table 4). Other preoperative and perioperative predictors were not significant for the recurrence of AR >  2+ during follow-up (Table 4). The cumulative freedom from all causes of cardiac reoperation was 94.2 ± 2.8% within the cohort at five years and 10 years (Fig. 1c). Aortic valve replacement was required in four patients with BAV morphology within a time frame of 11 to 36 months after initial surgery. Of these, two patients presented with symptomatic progressive severe AR and were reoperated due to the dehiscence of the direct suture line after the raphe triangular resection and one patient with disruption of the Gore–Tex suture after free-margin resuspension. One patient required aortic valve replacement due to infective endocarditis 25 months after the initial surgery. All patients successfully underwent mechanical aortic valve replacement with an uneventful postoperative course. Neither major bleeding nor thromboembolic or permanent neurologic events were found during follow-up.

**Table 4:** Univariate and multivariate (Cox regression) analysis for recurrent late AR (> 2+)

Variables	Univariate analysis Murtivariate analysis			
	HR (95% CI) *P* - value	HR (95% CI) *P* - value		
BAV	2.2 (0.75–6.7)	0.14		
AVJ ≥28 mm	1.3 (0.42–4.1)	0.62		
FMP FMR RZ Severe AR (≥3 +)	0.5 (0.1–1.6) 2.7 (0.92–8.0) 1.5 (0.39–6.2) 3.8 (0.24–61.7)	0.26 0.06 0.51 0.33		
eH < 9 (mm)	4.7 (1.3–16.6)	0.01	5.1 (1.3–19.1)	0.02
Residual mild AR (+ 1)	13.9 (4.2–45)	0.0001	24.9 (5.6–120)	0.0001

*AR* aortic regurgitation, *AVJ* aortoventricular junction, *BAV* bicuspid aortic valve, *CI* confidence interval, *eH* effective height, *FMP* free margin central plication, *FMR* free margin resuspension, *HR* hazard ratio, *RZ* triangular resection, *SI* sievers

## Discussion

The aortic valve reimplantation technique is a technically challenging procedure, and mostly performed for young and otherwise healthy patients with intact tricuspid AV and minimal AR to preserve the native AV and avoid valve replacement therapy [12]. Currently, the indications for AVS have been extended in high-risk patients with complex AR, particularly for individuals with congenitally determined AV morphology and aortopathy (connective tissue disorders) [13]. A thorough perception of the aortic root three-dimensional anatomy and functional interaction with AV cusps as an entire unit led to refinements and standardisation of AV repair sparing techniques [10]. Despite growing interest, primary ACR techniques are still debatable as fewer studies addressing this subject as more complex and less reproducible, especially in patients with severe preoperative AR, various phenotypes of the BAV, and reoperations [14]. Herein, we analysed our single institutional experience with AVS and associated ACR focusing on long-term outcomes and predictors of recurrent AR. David et al. [3] reported the longest available outcomes in a series of 333 patients (ACR in 64.4%). The mean age of the patiens and their clinical follow-up was 46 ± 15 years and 10.3 ± 6.8 years, respectively. In this cohort, the early mortality was 1.2% and the overall survival at 10 and 15 years was 89.5 ± 2.0% and 77.9 ± 3.9%, respectively. In a recent study, Mastrobuoni et al. [5] reported the long-term experience in 440 consecutive patients (mean age, 49 years; mean follow-up of five years; completeness of follow-up was 78%.) with aortic root aneurysm undergoing the reimplantation technique and a high number of associated ACR procedures (72.7%). The in-hospital mortality was 0.7% and the survival was 79.7% ± 3.8% at 10 years. In this study, we were able to maintain low mortality and morbidity in elective surgery for this young patients cohort with a normal comparable life expectancy; thereafter, in accordance with to most recently published reports [3–6]. Furthermore, De Kerchove et al. [2] reported that neither severe preoperative AR nor associated ACR had an impact on the durability of the AV repair and did not increase the risk of recurrent AR, in contrast to the findings in the meta-analysis conducted by Arabkhani et al. [15]. From the beginning of the AV repair sparing program, we performed more aggressive approaches combining AV reimplantation with ACR for bicuspid and tricuspid leaking AVs, who presented with significant preoperative AR (86.4%).

Moreover, a high percentage of the patients (83.9%) for type II AR dysfuntion underwent free-margin central plication as the most simple, reproducible, and efficient technique in case of cusp prolapse repair [3, 5].

Patients with chronic severe AR and dilated root might have frequently overstretched, thin, and prolapsing AV cusp tissue (type II AR dysfuntion) due to constant haemodynamic stress, thereby mandating extensive AV repair with different ACR techniques to reestablish competency of the aortic valve [1]. Other large AV sparing repair series (including the remodeling technique with external annuloplasty) demonstrated an increasing number of patients who required additional cusp repair due to unrecognised cusp disease or technically induced prolapse after reconstruction of the aortic root [5, 16].

Intraoperative TEE is an important tool in selecting patients for AV repair sparing surgery with guided specific reconstructive strategies and immediate evaluation of proper aortic valve performance after repair [17]. In 2009, several intraoperative predictors of recurrent AR after AV repair sparing surgery have been identified by le Polain de Waroux et al. [18] in retrospective study of 186 consecutive patients based on intraoperative TEE imaging. The main predictors have been described as follows: the presence of more than mild AR postoperatively, eccentric jet, coaptation below the annular plane, a coaptation length < 4 mm, and an enlarged aortic annulus. In our study, the multivariate analysis confirmed the tendency towards recurrent AR (> 2+) in patients with mild residual AR after surgery. However, this finding still has limited power to draw conclusions due to the relatively small number of events and needs other additional criteria for further evaluation. In recent years, Bierbach and Schäfers et al. [19] introduced the effective height concept *into clinical practice* as a prognostic predictor with the quantitative value to reestablish perfect valve configuration and long-term valve function. The effective height is measured and compared at equal distances beetwen cusps (from the middle of the free margin to the plane passing through the cusp insertion plane) intraoperatively by special calipers or with TEE evaluation [20]. Our study correlates well with findings from the Schäfers group *in terms of* low effective height value (< 9 mm), by multivariate analysis it was another independent predictor to induce recurrent AR > 2+ (p= 0.02). Therefore, a low effective height value after root reconstruction indicates residual cusp prolapse and mandates additional free-margin central plication in order to increase the coaptation height, as has been indicated in the literature [16]. We have applied free-margin central plication as our first choice; however, very aggressive prolapse correction in several instances was associated with recurrent AR due to restriction. At the beginning of our study, we tried triangular raphe resection (BAV type 1) with a direct suture; however, within the first years after initial surgery, AV failure presented and reoperations were necessary. Two patients experienced dehiscence of the direct suture line likely due to an inadequate quantitity of AV cusp tissue. In the literature, the lack of cusp tissue in BAV morphology is defined when the geometric height of the non-fused aortic cusp is 19 mm or less [21]. We stopped to employ this technique and changed our operative strategy to more consistent techniques (e.g., free margin central plication) to preserve tension-free repair and AV cusp mobility. All the components of the aortic root complex along with AV cusps have to be addressed and repaired with meticulous surgical techniques and comprehensive TEE evaluation to achieve excellent repair and long-term outcomes [22]. On the other hand, AV replacement with AV and composite root substitutes could be performed safely with less technical dificulty in patients with questionable AV quality and less than perfect AV reconstruction. In a recent study, Ouzounian et al. [23] reported comparative long-term outcomes and trends between patients undergoing AVS and AV root replacement surgery with composite valve grafts (mechanical and tissue) in patients with AR and aortic root pathology. The results of this analysis revealed that the rate of cumulative risk of valve-related complications increases after mechanical AV root replacement with time due to lifelong anticoagulation therapy (HR 5.6; p= 0.008), whereas tissue substitutes pose potential reoperation risk due to structural degeneration (HR 6.9; p= 0.003) compared with AVS surgery [23]. In contrast, the AV sparing repair surgery may be a considerable alternative to avoid prosthetic valve-related complications and lifelong anticoagulation therapy for active patients seeking normal life expectancy. Our analysis confirmed the low rates of valve-related complications (reoperation, thromboembolism, bleeding, infective endocarditis) without the need of lifelong anticoagulation therapy as reported by Saczkowski et al. [24] in a systematic review of AV preservation and repair. We believe that our data supplement existing outcomes and support the current trend towards extention of the indications for the AV sparing repair surgery for higher-risk patients rather than conventional replacement therapy; however, there is a lack of data directly comparing the AV sparing repair surgery to replacement.

### Limitations

This single-centre study is limited by its retrospective design. Certainly, heterogeneity of the patients, different ACR techniques, as well as the accumulation of surgical experience with the inevitable phase of the learning curve had major implications for reproducibility and clinically relevant outcomes. Continued follow-up and further standardised reporting are necessary to draw definitive conclusions for this challenging patient population.

## Conclusions

AVS with ACR, combined in a systematic fashion, is a safe and reproducible option with low risk of long-term valve related events and normal life expectancy for patients with pure aortic regurgitation.

The competent aortic valve and effective height, not lower than 9 mm intraoperatively, are mandatory to achieve long-lasting AV competency.

## Data Availability

The data generated or analysed during this study in this published article are available from the corresponding author on reasonable request.

## Abbreviations

ACR: Aortic cusp repair
AR: Aortic regurgitation
AV: Aortic valve
AVJ: Aortoventricular junction
AVS: Aortic valve sparing surgery
BAV: Bicuspid aortic valve
eH: Effective height
NYHA: *New York Heart Association* functional classification
STJ: Sinotubular junction
TEE: Transoesophageal echocardiography
